# Silent witness: a moss provides important evidence in solving a cemetery crime

**DOI:** 10.1093/fsr/owaf038

**Published:** 2026-03-05

**Authors:** Matt von Konrat, Llo Stark, Jenna Merkel, Anne Grauer, Wayne Jakalski, Paul Kiefer, Danny Kreider, Eric Leafblad, Alan Lichamer, Gary Merrill, Jason Moran, Gavin Quinn, Doug Seccombe, Kathryn Sodetz, Matthew Thrun

**Affiliations:** Department of Botany, The Field Museum, Chicago, IL, USA; School of Life Sciences, University of Nevada, Las Vegas, NV, USA; Department of Forensic Sciences, George Washington University, Washington, DC, USA; Department of Anthropology, Loyola University, Chicago, IL, USA; Cook County State's Attorney’s Office, Chicago, IL, USA; Public Corruption & Financial Crimes Unit, Cook County State’s Attorney’s Office, Chicago, IL, USA; Department of Botany, The Field Museum, Chicago, IL, USA; Cook County State's Attorney’s Office, Chicago, IL, USA; Kane County State’s Attorney’s Office, Chicago, IL, USA; Department of Botany, The Field Museum, Chicago, IL, USA; Department of Botany, The Field Museum, Chicago, IL, USA; Commander Special Victims/Forensic Services Division, Cook County Sheriff’s Police Department, Maywood, IL, USA; Internet Crimes Against Children Task Force, Cook County State’s Attorney’s Office, Chicago, IL, USA; Retired Special Agent, Federal Bureau of Investigation (FBI) Chicago Field Office, Chicago, IL, USA; Cook County State's Attorney’s Office, Chicago, IL, USA; Cook County State's Attorney’s Office, Chicago, IL, USA

**Keywords:** forensic sciences, biology, botanical evidence, botanical forensics, bryophytes, cemetery crime, mosses, prosecution

## Abstract

Forensic botany, particularly bryophyte analysis, is a potentially important but underutilized tool in criminal investigations. Bryophytes, including mosses, liverworts, and hornworts, have the potential to offer crucial evidence in establishing crime scene timelines and possible connections between suspects, victims, and the scene of the crime. In this case study, a common moss, *Fissidens taxifolius*, played a pivotal role in revealing the duration of the desecrated human remains and potential evidence for the location from which they had been disinterred. This case study highlights the need for greater utilization of forensic botany, especially microscopic plant material for its potential broader inclusion in criminal investigations.

**Key Points**
 

- Forensic botany: bryophytes, a potentially helpful forensic evidence tool, provide critical information in criminal cases.
- Widespread but overlooked: bryophytes’ ubiquitous nature and properties present great potential in forensic investigations, yet they are often overlooked.
- Key timeline evidence: bryophyte fragments found with buried human remains were used to develop a timeline, and played a pivotal role in reconstructing events.

## Introduction

Plant evidence is a vital yet underutilized tool within forensic science [1]. Information derived from botanical samples can be invaluable in drawing connections between suspects, victims, and crime scenes. Bryophytes, including mosses, liverworts, and hornworts, have been included as evidence in several criminal investigations and are reviewed in this article [2]. Forensic botany was even featured in the BBC television series “Silent Witness”, in which an episode included evaluation of the moss *Weissia rostellata* (Brid.) Lindb. This forensic botanical example inspired the study’s title. Bryophytes have several features and attributes that may serve as or corroborate other evidence [3]. Their abundance in diverse habitats and predictable response to ecological conditions make them reliable indicators of timelines and environmental changes [3, 4]. In this case study, we examine a crime that took place at Burr Oak Cemetery, Illinois, USA [5], where the desecration of burial sites led to the discovery of human remains in close association with bryophytes. This case highlights how bryophyte evidence was instrumental in establishing a timeline of the criminal activity, which eventually led to the prosecution and conviction of the suspects. This was the first instance of bryological evidence being used to establish a timeline in a courtroom in the state of Illinois in the USA.

## Case report

In 2009, Burr Oak Cemetery, a historic 150-acre graveyard in the Chicago suburb of Alsip, Illinois, was declared a crime scene after evidence of a scheme involving the removal of human remains and the reselling of existing burial plots was discovered [6, 7]. Burr Oak Cemetery holds profound historical significance in serving the African-American Community, as it contains the final resting place of Emmett Till, whose murder became a catalyst for the civil rights movement and a stark symbol of racial injustice in America. Four suspects were charged with illegally disinterring human remains and caskets, depositing the burials in an unused area of the cemetery, and selling the burial plots to families of recently deceased individuals [7]. Prosecutors asserted, after forensic investigation, that ~1 500 bones of at least 29 people had been illegally deposited on cemetery grounds [7, 8]. In May 2024, additional human remains attributed to this crime were discovered [9]. This crime led to the creation of the Bereaved Consumer’s Bill, introduced to Congress in 2011 [10].

During the investigation, within an area designated Crime Scene A, Feature 1, two types of plant material (a tree root and a bryophyte) were discovered in association with human skeletal remains. Investigators collected and documented the evidence for further analysis. Dr. Anne L. Grauer, the forensic anthropologist who assisted in the recovery of the plant samples from the cemetery, suggested that further analysis of the moss and tree roots might reveal the original location of the plants within the cemetery and shed light on the timeline of their deposition within the crime scene.

A moss specimen designated as Item No. 59 was identified as *Fissidens taxifolius* Hedw., and became key evidence in challenging the suspects’ defence. By determining the moss’s condition (e.g., physical appearance, health, stress) and its environment (e.g., abiotic factors, including light and moisture), investigators were ultimately able to establish that the desecration had occurred within 12 months, contradicting the suspects’ alibis and timeline of events.

## Materials and methods

### Evidence collection

Law enforcement divided the crime scene into four areas, each containing mounds of dirt above the ground surface [11]. Forensic anthropologists, the Cook County Sheriff’s Police Department (CCSPD) Evidence Technicians, and Federal Bureau of Investigation (FBI) Special Agents excavated the mounds. The plant specimens and bones were photographed, documented, and preserved for analysis.

Item No. 59, the moss sample, was placed in a clear plastic bag and shielded from sunlight before being transferred to a clean brown paper evidence packet for long-term storage. The samples were kept in dark, dry conditions to preserve their structural integrity until further botanical analysis could be performed [11].

### Herbarium analysis and identification

The Field Museum of Natural History is home to over 3.2 million dried plant specimens and played a critical role in identifying Item No. 59. Herbaria are vitally important sources of information about plants and the world they inhabit [12]. Additional herbarium specimens of the same species collected in the same county as the cemetery were used for reference; these are listed below. Experts specializing in the moss genus *Fissidens* were also consulted for their opinions on the identification of all specimens.

### Voucher specimens

The following list of voucher specimens, including Item No. 59, were used for comparison and are housed at Field Museum of Natural History (F):

*Fissidens taxifolius*, D. Hall, 2060, Cladwell Woods Golf Course Woods, Chicago, Cook County, IL, USA, https://fm-digital-assets.fieldmuseum.org/460/304/C1064784F.jpg [C1064784F];

*Fissidens taxifolius*, M. von Konrat, D. Seccombe and N. Trutenko, 24-08-01, Burr Oak Cemetery, 4400 W 127th St, Alsip., Cook County, IL, USA, https://collections-botany.fieldmuseum.org/catalogue/3565467 [C0308980F];

*Fissidens taxifolius*, Brian S. Clark, Item No. 59, Federal Bureau of Investigation (FBI), Burr Oak Cemetery, 4400 W 127th St, Alsip., Cook County, IL, USA, https://fm-digital-assets.fieldmuseum.org/1453/336/C0308979F.jpg [C0308979F], https://fm-digital-assets.fieldmuseum.org/1471/012/C0320394F.jpg [C0320394F];

*Fissidens taxifolius*, D. Hall, 2029, St. Pauls Woods, Morton Grove, Cook County, IL, USA, https://fm-digital-assets.fieldmuseum.org/460/313/C1064793F.jpg [C1064793F];

*Fissidens taxifolius*, D. Hall, 2006, Frank Bobrytzke Memorial Woods, Niles, Cook County, IL, USA, https://fm-digital-assets.fieldmuseum.org/460/305/C1064785F.jpg [C1064785F];

*Fissidens taxifolius*, D. Hall, 52, Beck Lake Woods Forest Preserve, Central road, Cook County, IL, USA, https://fm-digital-assets.fieldmuseum.org/1932/302/C0307224F.jpg [C0307224F].

### Crime scene visits and climatic data

On 11 August 2009, the crime scene and Burr Oak Cemetery were surveyed for the moss genus *Fissidens* and to assess the local ecology and environment. Jack Steed (Director, Financial Crimes/Public Corruption, Cook County Sheriff’s Department) provided climatic data spanning several years. The data, sourced from the US Department of Commerce National Data Center, were collected from a location 8.5 miles (13.68 km) from Burr Oak Cemetery. This information was crucial as climatic conditions may have impacted the physical nature of the evidence.

### Culturing and growth observations

To determine the relative viability and age of the samples, plants from the 2009 crime scene (Item No. 59), a herbarium specimen collected in 1995, and a fresh sample collected in July 2009 from Burr Oak Cemetery were cultured under controlled conditions. The protocol described by Stark et al. [13] was used, involving placing bryophyte samples in lidded watch glasses within a plant growth chamber under specific temperature, light, and humidity conditions. Researchers then regularly observed and recorded developmental stages and growth parameters.

### Measuring chlorophyll fluorescence

Although several physiological indicators can be considered to assess moss vitality, chlorophyll *a* fluorescence in leaves was monitored as an important physiological trait since it is considered a direct proxy for cellular damage within plant tissue [14]. The unit *F*_v_/*F*_m_ is a measure of the photosynthetic efficiency of plants and provides a reliable indicator of plant health and stress. *F*_v_/*F*_m_ is the ratio of variable fluorescence (*F*_v_) to maximum fluorescence (*F*_m_). Measurements were taken based on Barker et al. [15]. Two readings were recorded from the 1995 and 2009 plants, and one from Item No. 59 plants (three shoots each).

## Results

### Species identification and survey of the crime scene

**Figure 1:**
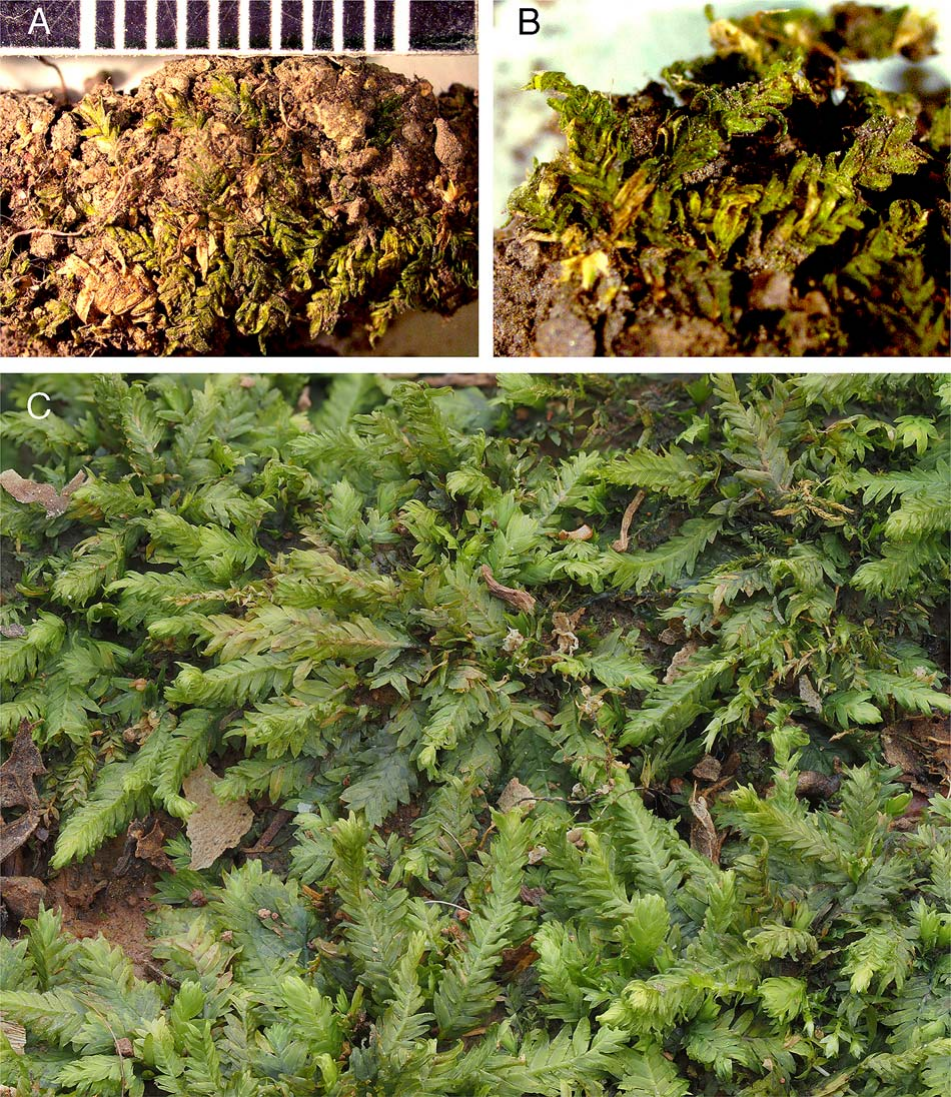
(A, B) The moss evidence (Item No. 59) that was discovered beneath the soil with human remains (scale in millimetres). (C) Typical habit of *Fissidens taxifolius* for comparison (photo copyright Blanka Aguero; reproduced with permission).

Item No. 59 (Figure 1A and B) was initially identified as belonging to the moss *F. taxifolius*. This species typically grows on soil and is globally distributed, being especially widespread in the Northern Hemisphere [16, 17]. *Fissidens* is one of the largest and most diverse moss genera, with ~450 species currently recognized [18].

**Figure 2:**
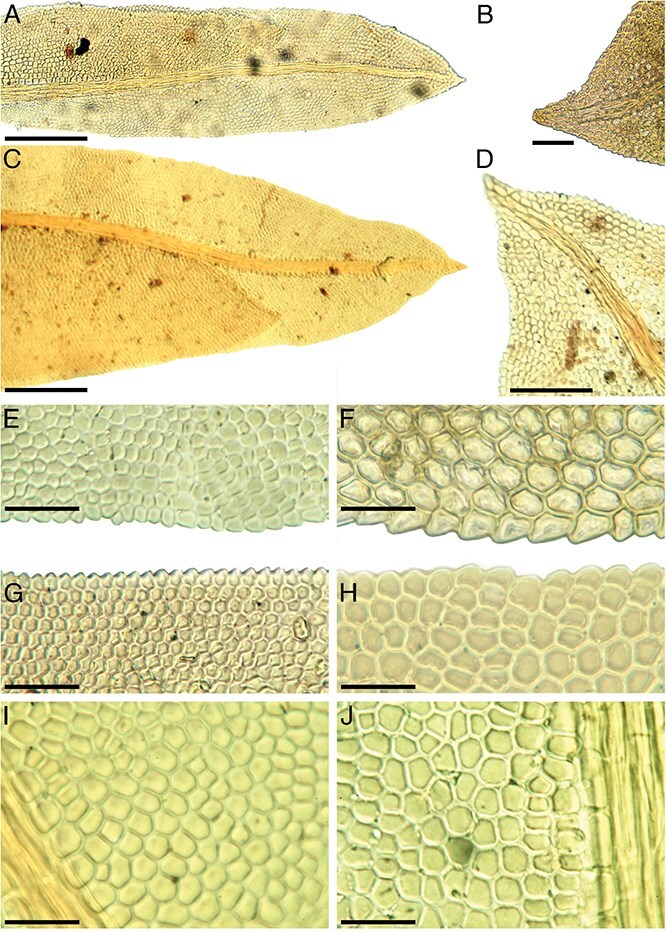
Leaves and cell anatomy of *Fissidens taxifolius*. Comparison of the evidence, Item No. 59 (C0308979F), with referenced material of *F. taxifolius*, Hall 2060, Cook County (C1064784F). Entire leaves and leaf apices: Item No. 59 (C0308979F) (A, B); Hall 2060 (C, D). Leaf margins: Item No. 59 (C0308979F) (E, F); Hall 2060 (G, H). Cell anatomy: Item No. 59 (C0308979F) (I); Hall 2060 (J). Scale bars: A, D = 100 μm; B, E, G = 50 μm; C = 200 μm; F, H, I, J = 25 μm.

A key diagnostic feature of the genus *Fissidens* is its unique leaf structure and arrangement. The leaves are two-ranked (“distichous”), aligned in two opposite rows along the stem (Figure 1C), giving the plant a distinctive flattened appearance. In *Fissidens*, each leaf is composed of three parts: a vaginant lamina, dorsal lamina, and ventral lamina (Figure 2A and C). Leaf shape, size, and margins are all used for species-level identification within the genus. Images of Item No. 59 and the specimens used for comparison in this study were shared with three internationally regarded experts on the genus *Fissidens,* who confirmed that they all belong to one species, *F. taxifolius.*  Figure 2 provides a comparison of the leaves, leaf margins, leaf apices, and leaf anatomy of Item No. 59 in comparison to a referenced specimen of *F. taxifolius*. Microscopic comparison confirmed that Item No. 59 is indeed *F. taxifolius*.

The crime scene and the cemetery were surveyed specifically for *Fissidens*. No plants belonging to this genus were found near the crime scene or in adjoining areas. This strongly indicated that Item No. 59 did not appear at the scene naturally by wind or animal activity. However, *Fissidens* was found in abundance near the margin of the cemetery in a lightly shaded area beneath trees amongst grave plots (Section 5, Lot 3). This was the same area from which law enforcement suspected illegal disinterment of human remains had originated. Subsequent examination confirmed plants in this area to be *F. taxifolius*.

### Estimating deposition of Item No. 59 in crime scene

The estimation of deposition duration for Item No. 59 was based on several factors: (i) decades of professional experience collecting and drying bryophytes in the field; (ii) expertise and knowledge of bryophytes, as well as deploying natural history collections as a point of reference; (iii) consultation with international colleagues; (iv) physiological experimental data from collections, including the evidence; (v) consideration of independent lines of evidence; (vi) bryological survey of the crime scene; and (vii) consideration of climatic data. It was hypothesized that if a moss plant was in the soil and in a dark environment, in combination with a high-moisture regime, the moss would soon die and decay. To test this theory, natural history collections were utilized, physiological experiments were conducted, and bryological expertise was pooled.

### Climatic data

In the 12 months prior to the summer of 2009, there was significant precipitation, including 5.8 inches (14.73 cm) in April and 6.8 inches (17.27 cm) in March of 2009, and 10.8 inches (27.43 cm) in September 2008. Water from this amount of rainfall would predictably penetrate 8 inches (20.32 cm) below the surface of the mound where Item No. 59 was discovered, suggesting that it had been exposed to periods of high levels of moisture. If the plants in the sample were still green and had not decayed appreciably, it might indicate that their burial duration was short.

### Viability, culturing, and fluorescence

**Figure 3:**
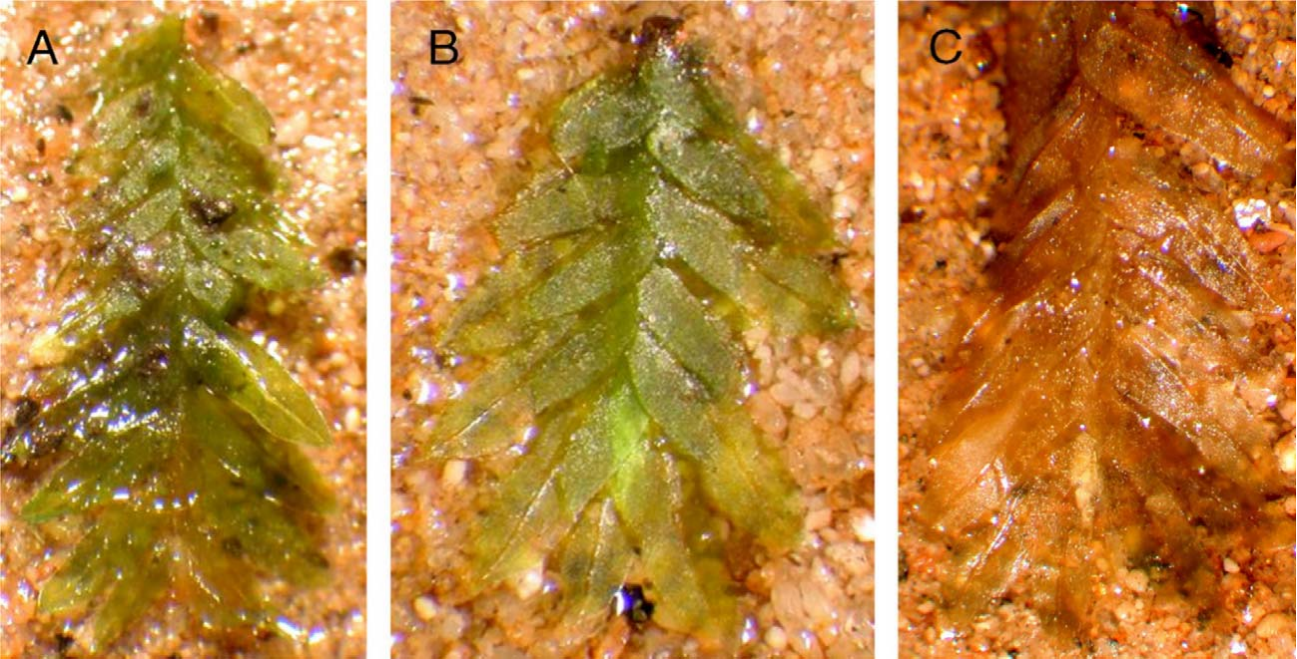
Samples from three differently aged specimens cultured in standardized conditions following the methods described by Stark et al. [13]. (A) Item No. 59. (B) Fresh specimen collected from Burr Oak Cemetery in August 2009. (C) Herbarium specimen collected in 1995.

Figure 3 shows how the plants appeared after only a single day of hydration in culture. The freshly collected 2009 specimen (Figure 3B) and Item No. 59 (Figure 3A) clearly retained most, if not all, of their green coloration. The 1995 herbarium specimen (Figure 3C) appeared dead, which would be expected from a dried, 14-year-old herbarium specimen. After several days of observation, the 2009 specimens were regenerating and initiating growth, which would be expected considering the fresh nature of the material. Item No. 59 was regenerating weakly, indicating that much of the tissue was dead, but with a few cells still alive.

**Figure 4:**
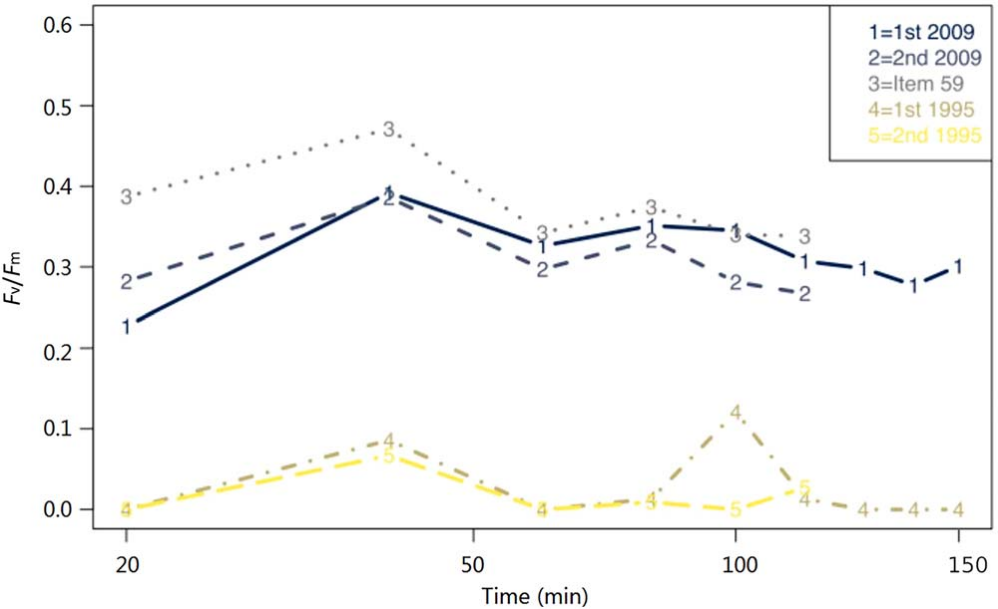
A graph comparing *Fissidens taxifolius* five samples over time. Lines 1 and 2 indicate two samples collected in 2009 representing fresh material; line 3 indicates the evidence, Item No. 59; and lines 4 and 5 indicate two samples collected in 1995. The varying lengths of dashes and solid lines and the varying colours aid the distinction between the five samples. The unit *F*_v_/*F*_m_ is a measure of the photosynthetic efficiency of the plants, which is a reliable measure of plant health and plant stress. Item No. 59 tracks very closely with the freshly collected material of 2009, at the time.

Figure 4 provides a comparison of the *F*_v_/*F*_m_ measurements between Item No. 59, 2009, and 1995 plants. The results from the chlorophyll fluorescence indicated that both Item No. 59 and the 2009 specimen were still viable. In contrast, the *F*_v_/*F*_m_ measurements clearly show the 1995 sample was not viable, which corroborates the coloration tests from the culturing.

### Independent line of evidence

Dr. Gary Watson (Head of Research, The Morton Arboretum), who was not involved in this study, suggested that the tree root found near the moss specimen and human remains was most likely severed from the tree within the last 1 or 2 years. This information was not known prior to the analysis and conclusions as outlined here, yet it further supports the conclusion as to the length of time that Item No. 59 was buried.

## Discussion

Three important requirements are necessary for bryophytes (and plants in general) to grow and/or survive: light, temperature, and moisture. Optimum conditions associated with these factors lead to growth. Extremes in any single factor typically lead to dormancy in some species, whereas combinations of extremes can lead to the death of plant tissue [19–21]. Light is essential in all plants, including bryophytes, as it is the energy source used by plants for photosynthesis. If environmental conditions are dry, many bryophytes are desiccation tolerant, and capable of reviving from an air-dry state [22]. However, if environmental conditions include a combination of high moisture and the absence of light (inhibiting photosynthesis), this will inevitably lead to death of plant tissue.

Analysis of the moss specimen (Item No. 59) recovered from the crime scene, when compared with fresh samples collected from surrounding areas, provided a critical timeline in support of the successful prosecution of this case. It demonstrated that Item No. 59 had been buried for no more than 24 months. In court proceedings of this case in 2015, the prosecution discredited the suspect’s claims that the newly buried individuals dated prior to their period of employment. A Frye Standard proceeding [23]—used in some US states, such as Illinois, to ensure the validity of a science and expert witness’s testimony introduced as evidence in court—was conducted to support the reliability and authenticity of bryology in this legal case. The inclusion of Item No. 59, alongside the analysis of the duration of deposition, was key for the prosecution during the Frye hearing. According to the prosecution’s research, moss had never been used in Illinois to establish a timeline for a crime. The prosecution conducted a Frye hearing to demonstrate that the lead author’s expert opinions, along with their consultation with experts during a workshop at an international meeting, constituted reliable and acceptable scientific evidence.

The combined analyses and observations in this study strongly indicated that Item No. 59 was a live plant when buried. It was likely exposed to no more than three seasons (spring/summer/fall) within a single year, predicated on the fact that the moss retained its green coloration, was shielded from sunlight within a soil mound above ground surface, and was saturated with infiltrating rainwater during the months of high precipitation. These facts supported the assertion that Item No. 59 had been deposited after the winter of 2007—during the employment of the suspects.

## Conclusion

The use of bryophytes in the Burr Oak Cemetery criminal investigation provides compelling evidence of their forensic potential. This investigation demonstrates how combining botanical identification and physiological experiments can yield crucial insights to assist forensic casework. It is clear that there is significant potential for bryophytes to be used within forensic science and that they, along with other plant material, especially microscopic, have possibly been underutilized. Undoubtedly, there are possible limitations to the applicability of bryophytes to forensic science, in terms of species-specific responses to biotic and abiotic factors. For example, different moss species may have varying responses to environmental conditions, as well as potential difficulties in preservation and analysing moss evidence in diverse environments. Yet, physical evidence is often presented in court, even where its value is limited, to corroborate witnesses or other physical evidence. We hope this encourages an increased awareness of bryophytes and similar microscopic plants when undertaking forensic investigation, ensuring critical plant evidence is not overlooked in the future.
